# Noninvasive Optoacoustic Imaging of Oxygen Saturation Reveals Hypoxic Hematopoietic Bone Marrow during Systemic Inflammation

**DOI:** 10.1021/acs.nanolett.5c01802

**Published:** 2025-10-01

**Authors:** Ashish Tiwari, Narmeen Haj, Ruth Pikovsky, Shirly Hagay, Maria Berihu, Betsalel Elgrably, Liron McLey, Majd Machour, Shiri Karni-Ashkenazi, Inbar Brosh, Shy Shoham, Shulamit Levenberg, Daniel Razansky, Amir Rosenthal, Katrien Vandoorne

**Affiliations:** † Faculty of Biomedical Engineering, 26747 Technion−Israel Institute of Technology, Haifa 3200003, Israel; ‡ NYU Langone Health, Tech4Health and Neuroscience Institutes, and Department of Ophthalmology, New York, New York 10016, United States; § Faculty of Medicine, University of Zurich, Zurich 8057, Switzerland; ∥ Department of Information Technology and Electrical Engineering, 31050 ETH Zurich, Zurich 8093, Switzerland; ⊥ Faculty of Electrical Engineering, 26747 Technion−Israel Institute of Technology, Haifa 3200003, Israel

**Keywords:** Optoacoustic imaging, Inflammation, Bone marrow, Lipopolysaccharides, Hypoxia, Oxygenation

## Abstract

Inflammation drives various diseases, including cardiovascular, neurodegenerative, and oncological disorders, by altering immune cell dynamics in hematopoietic niches. The bone marrow is the primary site for hematopoietic stem and progenitor cell activity. Here, we present a novel, noninvasive approach using multispectral optoacoustic tomography (MSOT) to track oxygenation dynamics in the murine calvarial bone marrow during acute systemic inflammation induced by lipopolysaccharide (LPS). Our MSOT system provided real-time, label-free imaging of hemoglobin oxygen saturation (sO_2_), revealing significant reductions in sO_2_ levels in lipopolysaccharide-treated mice, indicative of increased oxygen consumption. Co-registration with microCT enabled precise vascular mapping. Hypoxia was confirmed by ex vivo Pimonidazole staining and optical imaging and was associated with elevated neutrophil counts and enhanced hematopoietic activation. These findings demonstrate MSOT’s potential for noninvasive imaging of marrow oxygenation, offering insights into inflammation-driven hematopoietic activation and supporting the development of therapies targeting oxygen-sensitive pathways.

## Graphical abstract

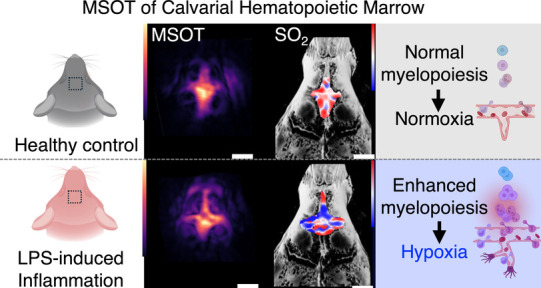

Inflammation drives the progression of numerous diseases, including cardiovascular disorders, diabetes, cancer, and neurodegeneration.^1^ Innate immune cells, such as neutrophils, are rapidly produced from hematopoietic stem and progenitor cells (HSPCs) in bone marrow niches in response to systemic inflammation.^2,3^ During inflammatory stress, HSPCs proliferate and differentiate into myeloid cells, a process tightly regulated by vascular signals.^4–6^ However, the bone marrow’s response can vary significantly, ranging from insufficient to overactive, critically impacting recovery and survival outcomes. Understanding the bone marrow response is especially important in conditions like sepsis, where the mortality rate is closely associated with the levels of circulating innate immune cells.^1^

The bone marrow vasculature supports hematopoiesis but also becomes hypoxic during heightened HSPC proliferation due to increased oxygen demand.^6,7^ Existing hypoxia detection methods, including Pimonidazole staining and two-photon phosphorescence microscopy, are invasive, while PET and MRI-based approaches suffer from low resolution or require exogenous tracers.^8–14^

Optoacoustic tomography provides a label-free, high-resolution alternative to assess oxygenation by measuring oxy- and deoxyhemoglobin levels.^15–17^ Here, we introduce multispectral optoacoustic tomography (MSOT) to noninvasively monitor oxygenation dynamics in murine calvarial bone marrow during systemic inflammation. This approach enables real-time tracking of hypoxia-driven hematopoietic activation, offering new insights into inflammatory responses originated from the hematopoietic bone marrow.

## Establishing Noninvasive Optoacoustic Imaging of Blood Oxygen Levels at the Calvarial Bone Marrow

Noninvasive imaging of bone marrow oxygenation has long posed a challenge due to the inherent scattering and absorption of light by bone tissue.^18,19^ To assess hemoglobin oxygenation in the hematopoietic bone marrow, we used a customized MSOT system (Figure 1A, B). To evaluate the feasibility of MSOT for this application, we first performed phantom experiments, which confirmed that placement of the superficial cranial bone layer did not attenuate optoacoustic signals or distort spectral features (Figure S1). Fluorescent beads mimicking hemoglobin absorption confirmed the system’s sensitivity and provided robust optoacoustic signal contrast in phantom studies (Figure S1A, B). Notably, placement of a superficial cranial bone layer above the beads did not attenuate the optoacoustic signal or alter its spectral characteristics, demonstrating signal preservation under tissue-like conditions (Figure S1C, D). HbO_2_ peaks at 532 and 576 nm, while Hb peaks at 560 nm (Figure 1C), enabling spectral unmixing of multiwavelength images. Co-registration with microCT provided precise vascular localization (Figure 1D–F, Figures S2 and S3). This demonstrated that MSOT can reliably penetrate the thin cortical layer of the murine calvaria,^20–22^ enabling robust and reproducible quantification of hemoglobin oxygenation within the hematopoietic marrow without the need for exogenous contrast agents. To improve anatomical resolution, we coregistered MSOT with microCT data, allowing precise spatial localization of marrow vasculature.^23^ Importantly, sO_2_ represents a localized, tissue-level hemoglobin oxygenation estimate based on optoacoustic spectral signatures and is different from systemic arterial oxygen saturation as measured by pulse oximetry.

**Figure 1:**
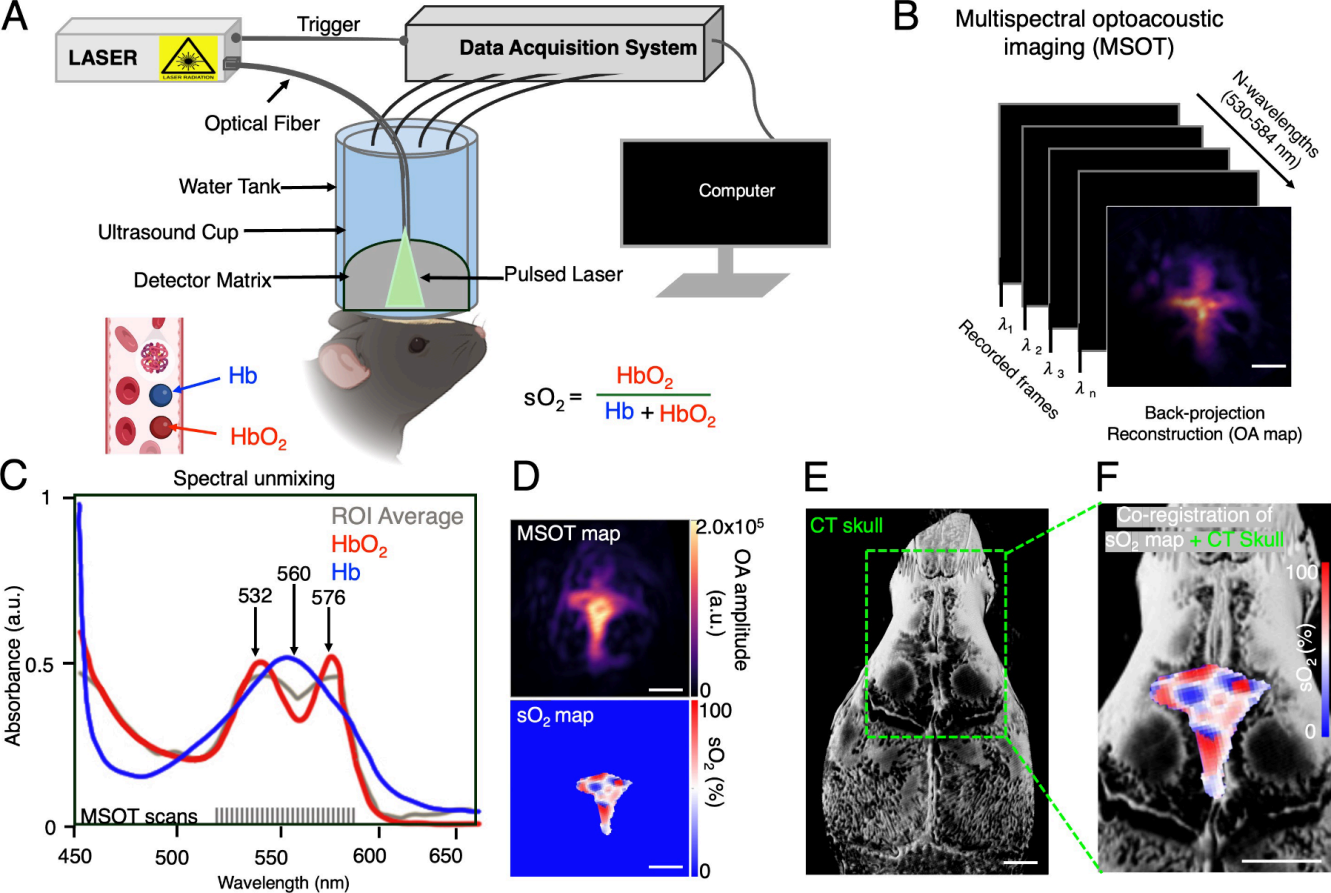
Multispectral optoacoustic tomography (MSOT) imaging setup for noninvasive oxygenation mapping in calvarial bone marrow. (A) The in vivo experimental setup for MSOT, to measure oxygenated (HbO_2_) and deoxygenated hemoglobin (Hb) and calculate the hemoglobin oxygen saturation (sO_2_). (B) Noninvasive MSOT imaging of the calvaria vessel at different wavelengths (530–584 nm). (C) Absorption spectra of HbO_2_ and Hb, along with the average signal spectra extracted from the MSOT data. (D) Representative vascular (single-wavelength) maximum intensity projection (MIP) images and MSOT-derived sO_2_ maps. (E) 3D-rendered microCT image of the mouse skull. (F) Co-registered MSOT and microCT images showing the spatial distribution of HbO_2_ within the calvarial bone marrow (scale bar = 1 mm).

Imaging anesthetized mice under oxygen versus air inhalation revealed a significant sO_2_ increase with oxygen (73.1 ± 7.1%) compared to air (52.1 ± 3.0%) (Figure 2A–C; Figures S4 and S5). These dynamic changes in MSOT-derived sO_2_ moving from controlled oxygen to air inhalation, validating the method’s sensitivity to physiological variation. To test imaging robustness, we compared MSOT-derived sO_2_ with and without the scalp intact using a skin-flap procedure procedure,^6,24,25^ finding no significant difference (sO_2_ with scalp: 52.1 ± 3.0%; sO_2_ without scalp: 52.3 ± 1.6%; Figure 2D–F; Figures S4 and S5), confirming the feasibility of noninvasive MSOT imaging. We further evaluated signal-to-noise ratio (SNR) and complementary deoxygenated hemoglobin (Hb) values under different experimental conditions to validate the reliability of our noninvasive imaging approach (Figure S5). Importantly, neither the type of inhaled gas nor the presence of overlying scalp tissue compromised signal quality or SNR, confirming the feasibility of fully noninvasive calvarial imaging. Collectively, these results support MSOT as a technically robust, repeatable modality for longitudinal assessment of marrow oxygenation. Our findings also extend previous work in bone marrow optoacoustic imaging. For example, Wood et al.^16^ successfully mapped sO_2_ in femoral bone marrow but encountered limitations due to the thicker cortical bone and the deeper anatomical location of the femur. In contrast, our calvarial approach benefits from minimal bone thickness and superficial location, enabling higher signal consistency.

**Figure 2:**
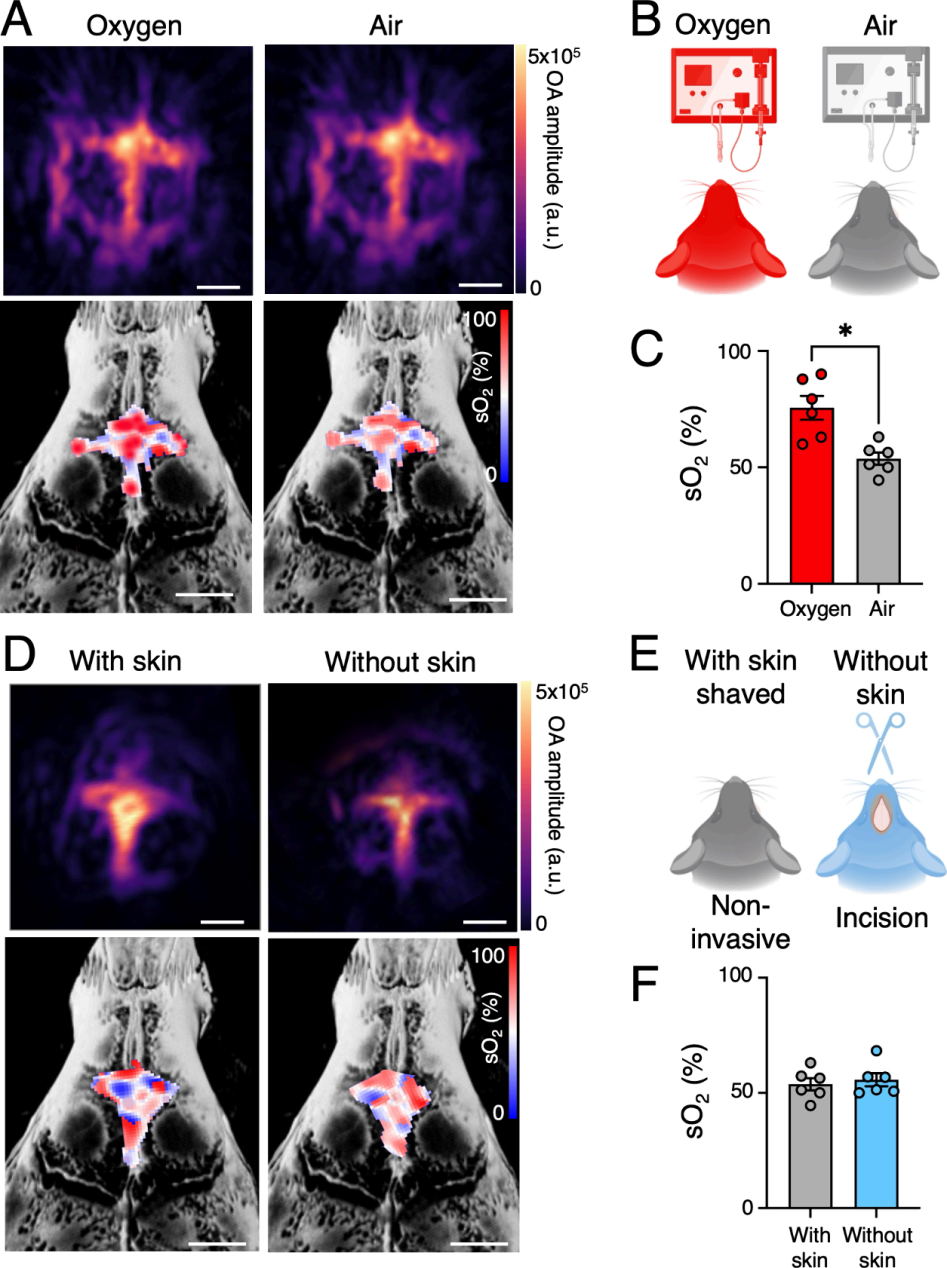
In vivo noninvasive and real-time MSOT mapping of hemoglobin oxygen saturation (sO_2_) at the calvarial hematopoietic marrow. (A) Representative vascular (single-wavelength) and unmixed sO_2_ images of calvarial hematopoietic marrow inhaled with oxygen and air (scale bar = 1 mm). (B) Schematic cartoon of the MSOT imaging experimental setup performed on healthy mice with an incised, open scalp inhaled with oxygen and air during isoflurane anesthesia. (C) sO_2_ values of calvarial marrow for oxygen and air inhaled mice. (D) Representative vascular and sO_2_ maps for calvarial hematopoietic marrow without skin and with skin (scale bar = 1 mm). (E) Schematics of the MSOT imaging experimental setup performed on healthy mice without opening the scalp (with skin) and with an incised, open scalp (without skin) inhaled with air during isoflurane anesthesia. (F) sO_2_ values of calvarial marrow for mice with and without a scalp. Real time data acquisition parameters: wavelength = 530–580 with an interval of 2 nm, speed of sound = 1480 m/s, lateral FOV = 10 × 10 mm, frequency filter = 0.1–6.0 MHz. (A Wilcoxon matched-pair signed-rank test was used for the statistical analysis; each dot represents one mouse; *P < 0.05; Scale bars: 1 mm).

Anatomical characterization of vascular structures in calvarial bone marrow. To characterize the vascular architecture of the calvarial bone marrow, we performed ex vivo microCT imaging of Microfil-perfused skulls (Figure 3A). We identified a highly vascularized diploic network running mediolaterally between the inner and outer cortical layers, with the interfrontal vein running dorsoventrally along the cranial suture (Figure 3B). Notably, axial microCT images of regions extending dorsally and ventrally from the marrow cavity revealed a shift in vessel depth, as the medial vein was observed at a deeper location befor and after the region of the calvarian marrow (Figure 3B). This shift highlights the dynamic anatomical organization of the vasculature across different regions of the skull. To characterize the vascular architecture of the calvarial bone marrow, histological and fluorescence microscopy confirmed that this vasculature is enclosed within an exceptionally thin cortical bone layer (Figure 3C–E), ensuring optimal optoacoustic signal transmission for MSOT-based imaging.

**Figure 3:**
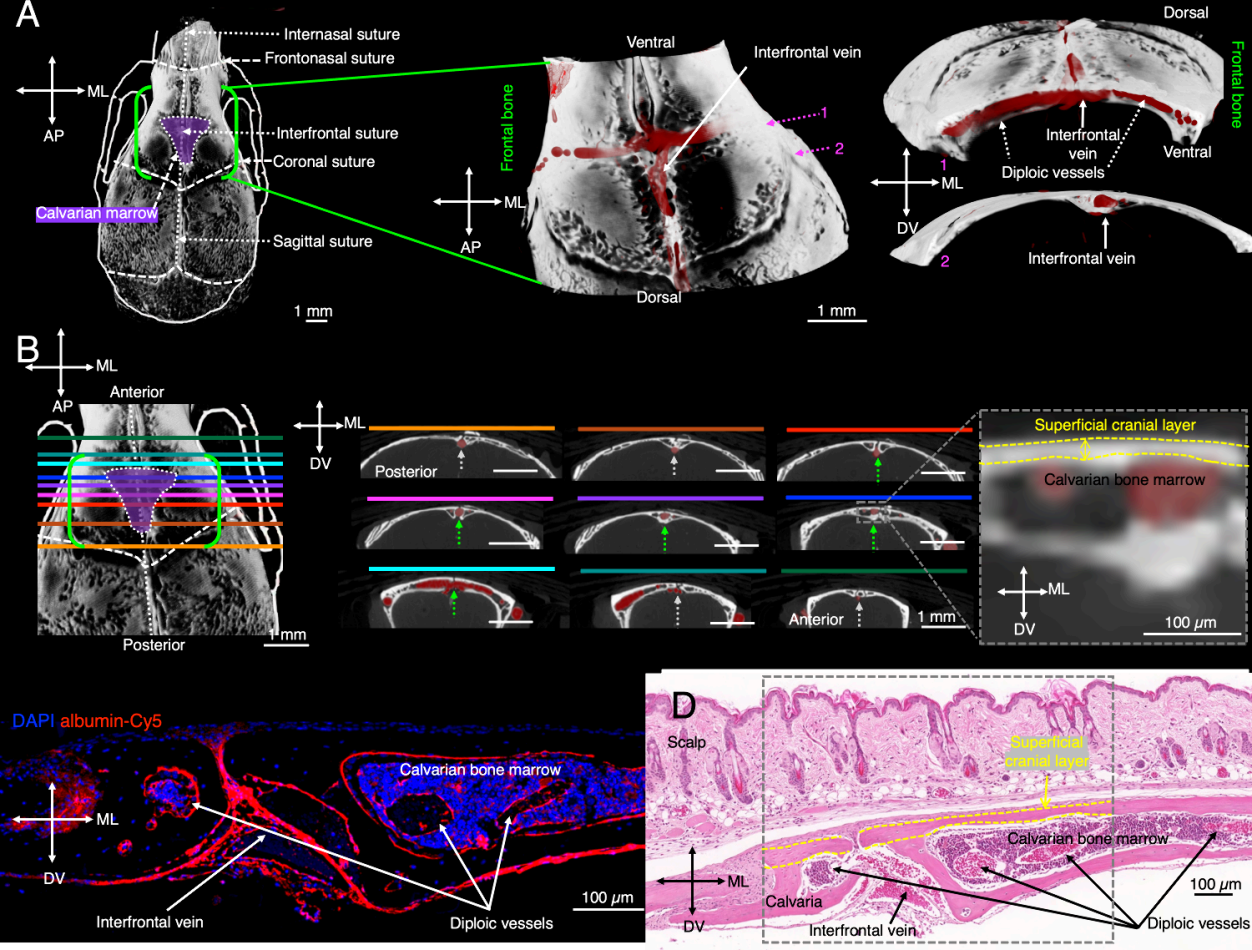
Visualization of calvarial bone marrow vessels and the interfrontal vein in mice. (A) A microCT scan of the mouse cranium highlights the frontal bone (outlined in green) and the calvarial bone marrow region (shaded in purple). Magnified three-dimensional (3D) reconstructions depict the bone structure in white and microfil-perfused vasculature in red. (B) Axial microCT images localize the interfrontal vein, positioned between the inner and outer layers of the calvarial bone in the bone marrow region (green dotted arrows). A zoomed inset (gray dotted square) illustrates the thickness of the superficial cranial layer (yellow dotted). (C) Ex vivo confocal microscopy of an axial section of the mouse skull reveals the calvarial bone marrow, with DAPI staining nuclei (blue) and albumin-Cy5 labeling vasculature (red). (D) Histological H&E staining of an axial cross-section of the skull reveals blood-filled vessels within the bone marrow, confirming their presence in this region. Arrows in panels (C–D) indicate the bone marrow, calvarial vasculature, and interfrontal vessels highlighting (gray dotted square) the exceptionally thin superficial cranial plate (yellow dotted) under the scalp, underscoring the accessibility for optoacoustic imaging. (Each dot represents 1 mouse, with 10 regions of interest averaged per mouse; the 3D visualization includes axes: MV = medioventral, AP = anteroposterior, and DV = dorsoventral).

## Acute Inflammation Reduces Calvarial Bone Marrow Oxygenation, As Visualized by Noninvasive Optoacoustic Imaging

To evaluate the effects of systemic inflammation on marrow oxygenation, we injected mice with lipopolysaccharide (LPS) and imaged them 18 h postinjection. Compared to controls, LPS-treated mice exhibited significantly lower sO_2_ levels in the calvarial bone marrow (sO_2_ in control: 52.05 ± 2.10%; sO_2_ in LPS: 40.52 ± 1.50%; *P* < 0.001) (Figure 4A–C, Figure S4). Unlike a previously studied leukemic model^16^—which may have introduced spatial heterogeneity in marrow oxygenation—our LPS-induced systemic inflammation model produced a more uniform hypoxic response, allowing us to detect clearer changes in sO_2_ and stronger alignment with hypoxia markers in histological validation. These findings also underscore the value of MSOT for longitudinal and noninvasive investigation of bone marrow physiology during inflammation. Inflammation drives emergency hematopoiesis, shifting the marrow microenvironment toward a metabolically active state.^25,26^ Therefore, the reduction in sO_2_ in LPS-treated mice revealed by MSOT imaging indicated increased oxygen consumption in the inflamed marrow. We additionally assessed SNR and corresponding hemoglobin values under control and LPS-treated conditions to confirm the reliability of our noninvasive imaging approach. SNR remained consistent between groups, indicating that systemic inflammation did not affect image quality (Figure S5C, F). We found that systemic inflammation induced by LPS did not compromise SNR. While intravital microscopy provides cellular resolution, it is limited to small fields of view (FOVs) and lacks intrinsic capability for quantifying oxygen saturation.^6,24,25,27^ MSOT, in contrast, allowed volumetric imaging of a larger calvarial bone marrow compartments and provided label-free functional readouts such as sO_2_.

**Figure 4:**
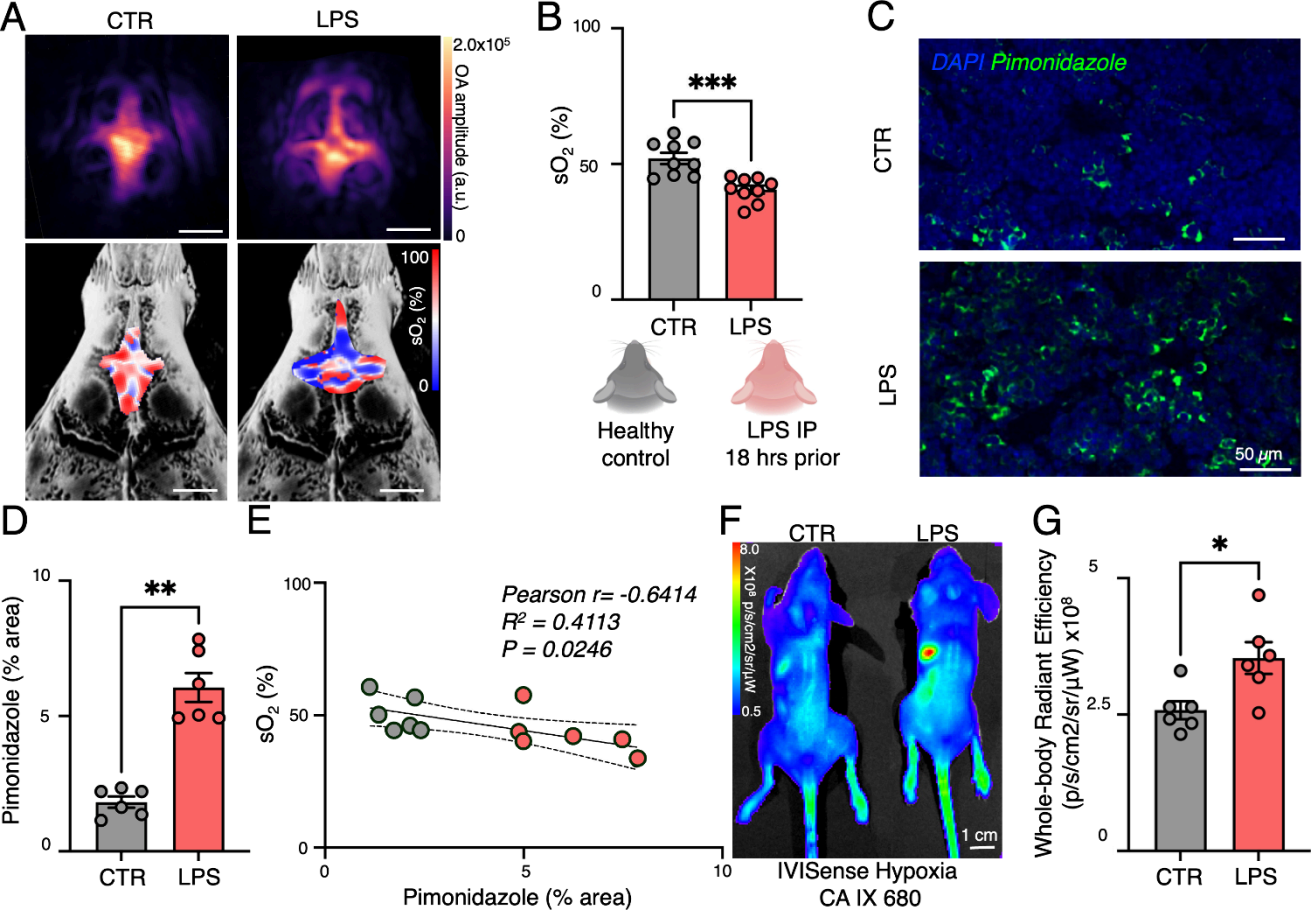
MSOT imaging reveals that systemic inflammation reduces oxygenation of the vascular bone marrow niche. (A) Representative vascular images and corresponding hemoglobin oxygen saturation (sO_2_) maps for control and LPS mice (scale bar = 1 mm). (B) sO_2_ values for control (CTR) mice and mice after LPS injection (LPS) at the calvarial hematopoietic marrow. MSOT imaging was performed noninvasively with shaved skin and air inhalation (5 regions of interest were averaged per mouse). Real time data acquisition parameters: 530–584 nm wavelength sweep with 2 nm steps, speed of sound 1480 m/s, FOV 10 × 10 mm, frequency cutoff 0.1–6.0 MHz. (C) Representative histological MIP images of the presence of Pimonidazole highlighting hypoxia in control (CTR) mice and mice after LPS injection (LPS). (D) Quantification of the Pimonidazole positive area in the metaphyseal bone marrow of the femur (*n* = 3 slices were stained per mouse; analyzed FOV = 250 × 250 μm). (E) Correlation between sO_2_ values and Pimonidazole-stained hypoxic area CTR and LPS bone marrow. (F) In vivo whole-body IVIS fluorescence imaging of IVISense Hypoxia CA IX 680 in CTR and LPS mice and (G) quantification of in vivo whole-body IVISense Hypoxia CA IX 680 signal. (A Mann–Whitney test was used for the statistical analysis; each dot represents one mouse; *P < 0.05; **P < 0.01; ***P < 0.001).

Following MSOT imaging, hypoxic regions were visualized using Pimonidazole staining, a well-established marker for detecting hypoxia. Pimonidazole staining of femoral bone marrow revealed expanded hypoxic regions in LPS-treated mice, confirming inflammation-induced hypoxia. A negative correlation (*r* = −0.6414, *R* ^2^ = 0.4113) between sO_2_ levels and the hypoxic area suggests that inflammation-driven metabolic shifts contribute to increased oxygen consumption in the bone marrow hematopoietic stem cell niche during systemic inflammation (Figure 4D, E). This strong inverse correlation (*r* = −0.6414) reinforces the physiological link between metabolic activity and the oxygenation status of the hemoglobin-containing hematopoietic niche, including capillaries, sinusoids, and small arteries.^25,27^

Using an orthogonal method, optical imaging with IVISense Hypoxia CA IX 680 further substantiated the presence of systemic bone marrow hypoxia during inflammation. After LPS injection, 2D whole body optical imaging revealed strong fluorescence signals in LPS-treated mice compared to control mice (Figure 4F, G). This pattern indicates that inflammation-induced hypoxia was not restricted to a localized site but was systemic, affecting multiple hematopoietic compartments. Ex vivo fluorescence analysis confirmed a notable increase in IVISense Hypoxia CA IX 680 fluorescent probe uptake in both the calvarial and femoral bone marrow of the LPS-treated mice compared to the signal observed in the control marrow (Figure S6). These findings align with our MSOT results and support the presence of systemic bone marrow hypoxia in this inflammation model. Importantly, the consistent results between MSOT and optical imaging strengthen confidence in our conclusion that systemic inflammation triggers widespread oxygen depletion, driving emergency hematopoiesis. These findings align with prior studies reporting hypoxia-driven hematopoietic activation in inflammatory conditions, such as sepsis,^6^ leukemia,^16^ and neuroinflammation.^22,26^

## Blood Oxygen Levels at the Calvarial Marrow Correlate with Blood Neutrophil Count and Hematopoietic Activation

To further assess the impact of reduced oxygenation, we analyzed hematopoietic activation and vascular remodeling. Complete blood count (CBC) analysis confirmed an increase in blood neutrophil levels in LPS-treated mice (Figure 5A). LPS-induced increase in circulating neutrophils, reflects previously reported enhanced myelopoiesis.^6^ Ex vivo fluorescence microscopy revealed expanded endomucin+ blood vessels and increased Ki67+ hematopoietic proliferation in the femoral marrow (Figure 5B–D). This aligns with known biological mechanisms where hypoxia often acts as a proliferative stimulus in various tissues.^28^ Oxygenation levels negatively correlated with vessel diameter (*r* = −0.8134, *R* ^2^ = 0.6616) and proliferating cell counts (*r* = −0.7468, *R* ^2^ = 0.5578) (Figure 5E, F), indicating that hypoxia promotes hematopoietic activation. The strong negative correlation between vascular expansion and oxygen levels suggests that inflammatory hypoxia induces vascular remodeling to support heightened metabolic demand.^6,29–31^ Intravital microscopy of Fluorescent Ubiquitination-based Cell Cycle Indicator (FUCCI)+ cells confirmed a significant increase in proliferating hematopoietic cells after LPS injection (Figure 5G–H). This increase suggests that the proliferating bone marrow cells have a higher oxygen demand, which is consistent with reports of Toll-Like Receptor 4-mediated HSPC proliferation.^4,5^

**Figure 5:**
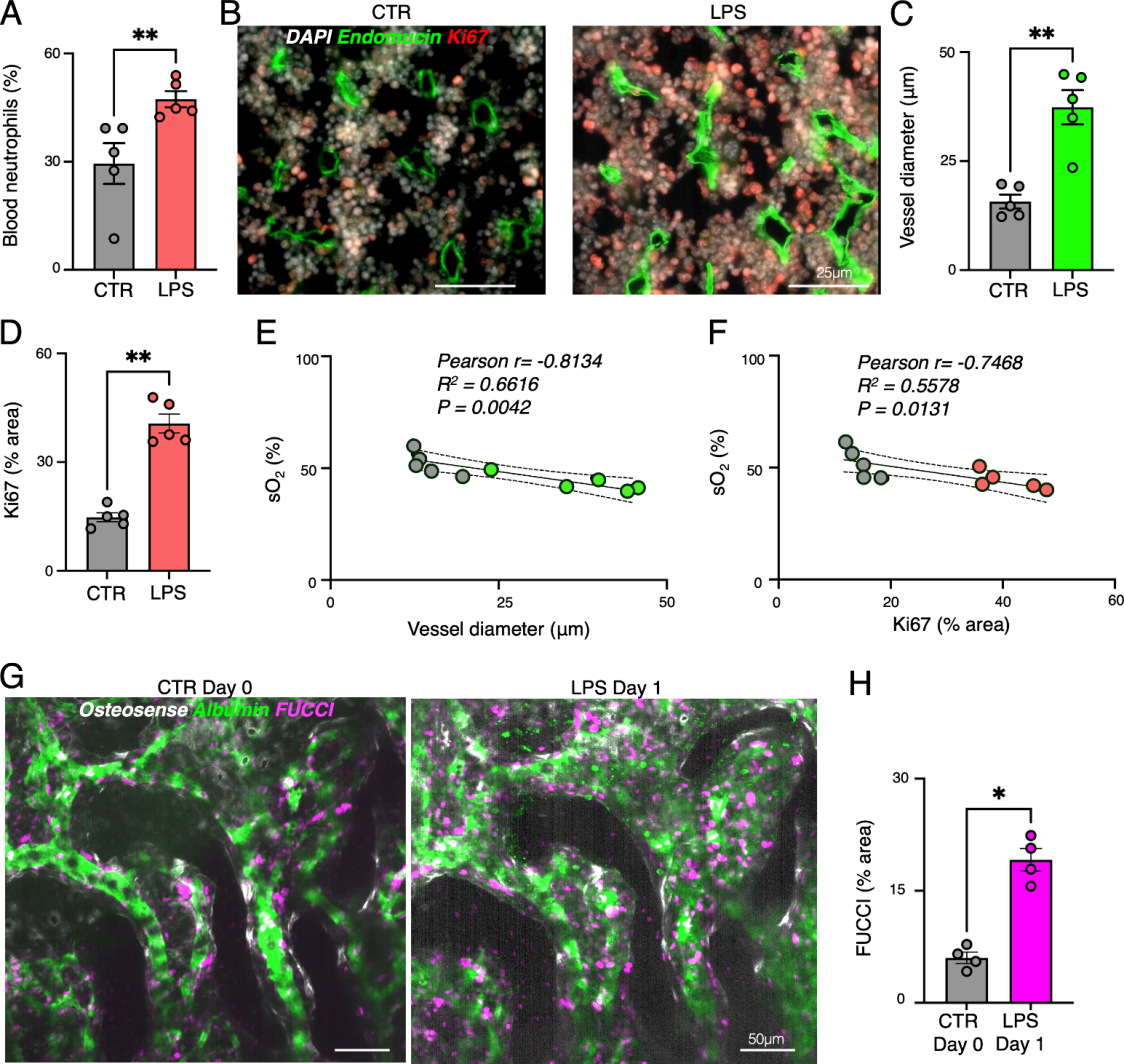
Lipopolysaccharide (LPS)-induced systemic inflammation increases vessel diameter and enhances proliferation in the hematopoietic bone marrow. (A) Neutrophil count studied by complete blood count analysis taken right after MSOT imaging. (B) Representative histological images of the presence of endomucin and Ki67 as detected by immunofluorescence in control (CTR) mice and mice after LPS injection. (C) Quantification of endomucin-positive vessel diameter in the metaphyseal bone marrow of the femur. (D) Quantification of Ki67+ area in the metaphyseal bone marrow of the femur (FOV = 250 × 250 μm). (E) Correlation between hemoglobin oxygen saturation (sO_2_) values and endomucin-positive vessel diameter in the metaphyseal bone marrow of the femur in CTR mice and mice after LPS injection. (F) Correlation between sO_2_ values and Ki67+ area in the metaphyseal bone marrow of the femur in CTR mice and mice after LPS injection. (G) Intravital confocal MIP images of osteosense^+^ calvarian bone marrow niches, dextran-FITC stained vessels, and FUCCI+ hematopoietic bone marrow cells in CTR mice at day 0 and in the same mice 1 day (18 h) after LPS injection (day 1; *n* = 4). (H) Quantification of FUCCI+ area (FOV = 250 × 250 μm). (A Mann–Whitney test was used for the statistical analysis; FOV = 250 × 250 μm; each dot represents one mouse (5 regions of interest were averaged per mouse); *P < 0.05, **P < 0.01).

This study establishes MSOT as a powerful tool for tracking bone marrow metabolism during inflammation. The ability to noninvasively quantify the bone marrow oxygenation status provides unique insights into bone marrow hypoxia and hematopoietic activation in real time, which could aid in monitoring disease progression in inflammatory and hematological disorders.^5,6,29–31^ Unlike prior noninvasive approaches such as BOLD-MRI, 18F-FAZA-PET, and 19F-MRI,^10–14^ which have been limited by low spatial resolution or the need for exogenous tracers, MSOT offers a label-free, high-resolution alternative.

A major translational challenge of MSOT imaging for bone marrow is bone thickness. While the murine skull is relatively thin,^32,33^ human bones are significantly thicker, posing a greater barrier to light penetration and optoacoustic signal generation. Future adaptations, such as longer-wavelength near-infrared light or acoustic wavefront correction techniques, may overcome these challenges.^34^ We acknowledge that transcranial optoacoustic imaging is prone to skull-induced acoustic distortions, as reported in prior studies.^18,19^ However, our imaging targeted the calvarial bone marrow - not the brain parenchyma- which resides between the thin inner and outer cortical layers of the skull. This anatomical location, combined with the minimal bone thickness of the outer cortical layer, allowed us to achieve robust and reliable optoacoustic signal acquisition without significant distortion. Future technological adaptations, such as longer-wavelength near-infrared light or acoustic wavefront correction methods,^34^ may help address these limitations for clinical translation.

Nonetheless, several limitations should be considered. (1) Light fluence correction was not applied, which may introduce minor errors in quantification.^35^ (2) Systemic hypoxia was assessed in the femur rather than the calvaria, assuming comparable responses across hematopoietic sites, though regional variability cannot be excluded.^6^ (3) While LPS is a well-established model of acute inflammation, it represents a specific bacterial endotoxin response; additional models such as cytokine-induced or sterile inflammation should be examined for broader relevance. (4) C57BL/6J mice were selected due to their widespread use in vascular and hematopoietic studies involving systemic inflammation and diabetes. Although pigmentation can attenuate optoacoustic signals at shorter wavelengths, we confirmed that the scalp remained unpigmented (pink) in this study, consistent with prior reports.^36^ (5) Recent developments in photoacoustic tomography^37,38^ offer exciting opportunities to expand this work and highlight the broader applicability of optoacoustic imaging for biomedical applications.

The bone marrow plays a central role in hematopoiesis, maintaining immune homeostasis through a specialized vascular network. Using a noninvasive, real-time optoacoustic imaging technique, we quantified oxygenation dynamics within the calvarial bone marrow during systemic inflammation. Our study introduces two key advancements: (1) a label-free MSOT approach to monitor hemoglobin oxygenation in the calvarial niche and (2) the ability to noninvasively track hypoxia-associated hematopoietic activation in real time. Importantly, these MSOT findings were corroborated by complementary readouts including Pimonidazole staining, intravital microscopy, circulating neutrophil counts, and optical hypoxia imaging, which together confirmed systemic hypoxia across multiple hematopoietic compartments.

This work establishes MSOT as a powerful noninvasive tool for longitudinal assessment of bone marrow physiology. We show that systemic inflammation induces profound metabolic shifts, characterized by bone marrow hypoxia, vascular remodeling, and emergency hematopoiesis. By integrating MSOT with histological, optical, and intravital methods, we provide a robust multimodal framework for investigating bone marrow responses under physiological and pathological conditions. The strong concordance between MSOT and optical imaging results underscores the reliability of our approach. Looking ahead, efforts should focus on optimizing MSOT for clinical translation, refining quantification methods, and extending its application to a broader range of inflammatory and hematological disorders.

## Supplementary Material



## Data Availability

The data that support the findings of this study are available on request from the corresponding author.

## Notes

I: Materials and methods; II: Additional figures; III: Supplementary references (PDF)

## #

Amity Institute of Nanotechnology, Amity University Noida, 201313, India

## References

[1] Furman D., Campisi J., Verdin E., Carrera-Bastos P., Targ S., Franceschi C., Ferrucci L., Gilroy D. W., Fasano A., Miller G. W., Miller A. H., Mantovani A., Weyand C. M., Barzilai N., Goronzy J. J., Rando T. A., Effros R. B., Lucia A., Kleinstreuer N., Slavich G. M. (2019). Chronic Inflammation in the Etiology of Disease across
the Life Span. Nature Medicine.

[2] Kopp H.-G., Avecilla S. T., Hooper A. T., Rafii S. (2005). The Bone Marrow Vascular
Niche: Home of HSC Differentiation and Mobilization. Physiology (Bethesda).

[3] Naveiras O., Daley G. Q. (2006). Stem Cells and Their Niche: A Matter
of Fate. Cell. Mol. Life Sci..

[4] Demel U. M., Lutz R., Sujer S., Demerdash Y., Sood S., Grünschläger F., Kuck A., Werner P., Blaszkiewicz S., Uckelmann H. J., Haas S., Essers M. A. G. (2022). A Complex Proinflammatory Cascade
Mediates the Activation of HSCs upon LPS Exposure in Vivo. Blood Adv..

[5] Nagai Y., Garrett K. P., Ohta S., Bahrun U., Kouro T., Akira S., Takatsu K., Kincade P. W. (2006). Toll-like Receptors
on Hematopoietic Progenitor Cells Stimulate Innate Immune System Replenishment. Immunity.

[6] Vandoorne K., Rohde D., Kim H.-Y., Courties G., Wojtkiewicz G., Honold L., Hoyer F. F., Frodermann V., Nayar R., Herisson F., Jung Y., Désogère P. A., Vinegoni C., Caravan P., Weissleder R., Sosnovik D. E., Lin C. P., Swirski F. K., Nahrendorf M. (2018). Imaging the
Vascular Bone Marrow Niche During Inflammatory Stress. Circ. Res..

[7] Rohde D., Vandoorne K., Lee I.-H., Grune J., Zhang S., McAlpine C. S., Schloss M. J., Nayar R., Courties G., Frodermann V., Wojtkiewicz G., Honold L., Chen Q., Schmidt S., Iwamoto Y., Sun Y., Cremer S., Hoyer F. F., Iborra-Egea O., Muñoz-Guijosa C., Ji F., Zhou B., Adams R. H., Wythe J. D., Hidalgo J., Watanabe H., Jung Y., van der Laan A. M., Piek J. J., Kfoury Y., Désogère P. A., Vinegoni C., Dutta P., Sadreyev R. I., Caravan P., Bayes-Genis A., Libby P., Scadden D. T., Lin C. P., Naxerova K., Swirski F. K., Nahrendorf M. (2022). Bone Marrow
Endothelial Dysfunction Promotes Myeloid Cell Expansion in Cardiovascular
Disease. Nat. Cardiovasc Res..

[8] Harutyunyan K. G., Nwajei F., Zal M. A., Fruman D. A., Mallampati S., Sun X., Zal T., Konopleva M. (2014). The Dynamics of Stroma-Leukemia Interaction
in the Hypoxic BM Niches in Vivo. Blood.

[9] Spencer J. A., Ferraro F., Roussakis E., Klein A., Wu J., Runnels J. M., Zaher W., Mortensen L. J., Alt C., Turcotte R., Yusuf R., Côté D., Vinogradov S. A., Scadden D. T., Lin C. P. (2014). Direct
Measurement
of Local Oxygen Concentration in the Bone Marrow of Live Animals. Nature.

[10] Roussakis E., Li Z., Nichols A. J., Evans C. L. (2015). Oxygen-Sensing Methods in Biomedicine
from the Macroscale to the Microscale. Angew.
Chem., Int. Ed..

[11] Perez R. C., Kim D., Maxwell A. W. P., Camacho J. C. (2023). Functional Imaging
of Hypoxia: PET and MRI. Cancers (Basel).

[12] Halmos G. B., Bruine de Bruin L., Langendijk J. A., van der Laan B. F. A.
M., Pruim J., Steenbakkers R. J. H.
M. (2014). Head and Neck Tumor
Hypoxia Imaging by 18F-Fluoroazomycin-Arabinoside (18F-FAZA)-PET:
A Review. Clin Nucl. Med..

[13] O’Connor J. P.
B., Robinson S. P., Waterton J. C. (2019). Imaging Tumour Hypoxia with Oxygen-Enhanced
MRI and BOLD MRI. Br J. Radiol.

[14] Eidelberg D., Johnson G., Barnes D., Tofts P. S., Delpy D., Plummer D., McDonald W. I. (1988). 19F NMR
Imaging of Blood Oxygenation
in the Brain. Magn Reson Med..

[15] Mitcham T., Taghavi H., Long J., Wood C., Fuentes D., Stefan W., Ward J., Bouchard R. (2017). Photoacoustic-Based
sO­(2) Estimation through Excised Bovine Prostate Tissue with Interstitial
Light Delivery. Photoacoustics.

[16] Wood C., Harutyunyan K., Sampaio D. R. T., Konopleva M., Bouchard R. (2019). Photoacoustic-Based
Oxygen Saturation Assessment of
Murine Femoral Bone Marrow in a Preclinical Model of Leukemia. Photoacoustics.

[17] Ni R., Straumann N., Fazio S., Dean-Ben X. L., Louloudis G., Keller C., Razansky D., Ametamey S., Mu L., Nombela-Arrieta C., Klohs J. (2023). Imaging Increased Metabolism in the
Spinal Cord in Mice after Middle Cerebral Artery Occlusion. Photoacoustics.

[18] Xu M., Wang L. V. (2006). Photoacoustic Imaging in Biomedicine. Rev. Sci. Instrum..

[19] Wang X., Pang Y., Ku G., Xie X., Stoica G., Wang L. V. (2003). Noninvasive Laser-Induced Photoacoustic
Tomography
for Structural and Functional in Vivo Imaging of the Brain. Nat. Biotechnol..

[20] Tabani H., Tayebi Meybodi A., Benet A. (2020). Venous Anatomy of the Supratentorial
Compartment. Handb Clin Neurol.

[21] White H. E., Goswami A., Tucker A. S. (2021). The Intertwined
Evolution and Development
of Sutures and Cranial Morphology. Front Cell
Dev Biol..

[22] Ni R., Chen Z., Deán-Ben X. L., Voigt F. F., Kirschenbaum D., Shi G., Villois A., Zhou Q., Crimi A., Arosio P., Nitsch R. M., Nilsson K. P. R., Aguzzi A., Helmchen F., Klohs J., Razansky D. (2022). Multiscale Optical and Optoacoustic
Imaging of Amyloid-β Deposits in Mice. Nat. Biomed Eng..

[23] Rosenblum J. S., Cappadona A. J., Lookian P. P., Chandrashekhar V., Bryant J.-P., Chandrashekhar V., Zhao D. Y., Knutsen R. H., Donahue D. R., McGavern D. B., Kozel B., Heiss J. D., Zhuang Z. (2022). Non-Invasive
in Situ Visualization of the Murine Cranial
Vasculature. Cell Rep. Methods.

[24] Lo
Celso C., Lin C. P., Scadden D. T. (2011). In Vivo Imaging
of Transplanted Hematopoietic Stem and Progenitor Cells in Mouse Calvarium
Bone Marrow. Nat. Protoc..

[25] Christodoulou C., Spencer J. A., Yeh S. C. A., Turcotte R., Kokkaliaris K. D., Panero R., Ramos A., Guo G., Seyedhassantehrani N., Esipova T. V., Vinogradov S. A., Rudzinskas S., Zhang Y., Perkins A. S., Orkin S. H., Calogero R. A., Schroeder T., Lin C. P., Camargo F. D. (2020). Live-Animal Imaging
of Native Haematopoietic Stem and Progenitor Cells. Nature.

[26] Deán-Ben X. L., Robin J., Nozdriukhin D., Ni R., Zhao J., Glück C., Droux J., Sendón-Lago J., Chen Z., Zhou Q., Weber B., Wegener S., Vidal A., Arand M., El Amki M., Razansky D. (2023). Deep Optoacoustic
Localization Microangiography of Ischemic Stroke in Mice. Nat. Commun..

[27] Li W., Liu Y.-H., Estrada H., Rebling J., Reiss M., Galli S., Nombela-Arrieta C., Razansky D. (2020). Tracking Strain-Specific
Morphogenesis and Angiogenesis of Murine Calvaria with Large-Scale
Optoacoustic and Ultrasound Microscopy. J. Bone
Miner Res..

[28] Yoon D., Ponka P., Prchal J. T. (2011). Hypoxia. 5. Hypoxia and Hematopoiesis.
American Journal of Physiology-Cell. Physiology.

[29] Boettcher S., Ziegler P., Schmid M. A., Takizawa H., van Rooijen N., Kopf M., Heikenwalder M., Manz M. G. (2012). Cutting Edge: LPS-Induced
Emergency Myelopoiesis Depends on TLR4-Expressing Nonhematopoietic
Cells. J. Immunol..

[30] Takizawa H., Fritsch K., Kovtonyuk L. V., Saito Y., Yakkala C., Jacobs K., Ahuja A. K., Lopes M., Hausmann A., Hardt W. D., Gomariz Á., Nombela-Arrieta C., Manz M. G. (2017). Pathogen-Induced
TLR4-TRIF Innate Immune Signaling
in Hematopoietic Stem Cells Promotes Proliferation but Reduces Competitive
Fitness. Cell Stem Cell.

[31] Li X., Tupper J. C., Bannerman D. D., Winn R. K., Rhodes C. J., Harlan J. M. (2003). Phosphoinositide
3 Kinase Mediates Toll-like Receptor
4-Induced Activation of NF-Kappa B in Endothelial Cells. Infect. Immun..

[32] Herisson F., Frodermann V., Courties G., Rohde D., Sun Y., Vandoorne K., Wojtkiewicz G. R., Masson G. S., Vinegoni C., Kim J., Kim D. E., Weissleder R., Swirski F. K., Moskowitz M. A., Nahrendorf M. (2018). Direct Vascular Channels Connect Skull Bone Marrow
and the Brain Surface Enabling Myeloid Cell Migration. Nature Neuroscience.

[33] Gulner B. R., Navabi Z. S., Kodandaramaiah S. B. (2022). 3D Morphometric Analysis of Mouse
Skulls Using Microcomputed Tomography and Computer Vision. bioRxiv.

[34] Scheeren T. W. L., Schober P., Schwarte L. A. (2012). Monitoring
Tissue Oxygenation by
near Infrared Spectroscopy (NIRS): Background and Current Applications. J. Clin Monit Comput.

[35] Zhou X., Akhlaghi N., Wear K. A., Garra B. S., Pfefer T. J., Vogt W. C. (2020). Evaluation of Fluence
Correction Algorithms in Multispectral
Photoacoustic Imaging. Photoacoustics.

[36] Li H., Fan L., Zhu S., Shin M. K., Lu F., Qu J., Hou L. (2017). Epilation
Induces Hair and Skin Pigmentation through an EDN3/EDNRB-Dependent
Regenerative Response of Melanocyte Stem Cells. Sci. Rep.

[37] Lv J., Lan H., Qin A., Sun T., Shao D., Gao F., Yao J., Avanaki K., Nie L. (2024). Dynamic Synthetic-Scanning Photoacoustic
Tracking Monitors Hepatic and Renal Clearance Pathway of Exogeneous
Probes in Vivo. Light: Science & Applications.

[38] Sun T., Lv J., Zhao X., Li W., Zhang Z., Nie L. (2023). In Vivo Liver
Function Reserve Assessments in Alcoholic Liver Disease by Scalable
Photoacoustic Imaging. Photoacoustics.

