# Supplementary material for: Noninvasive Optoacoustic Imaging of Oxygen Saturation Reveals Hypoxic Hematopoietic Bone Marrow during Systemic Inflammation
Source: Nano Lett. 2025 Oct 1;25(41):14767–75. doi: 10.1021/acs.nanolett.5c01802 (PMC12893716; doi:10.1021/acs.nanolett.5c01802)
Supplement: Supplementary file 1 [file nl5c01802_si_001.pdf]

Supporting Information for

Non-invasive Optoacoustic Imaging of Oxygen Saturation Reveals Hypoxic Hematopoietic Bone Marrow during Systemic Inflammation

*Ashish Tiwari [a], Narmeen Haj [a], Ruth Pikovsky [a], Shirly Hagay [a], Maria Berihu [a], Betsalel Elgrably [a], Liron McLey [a], Majd Machour [a], Shiri Karni-Ashkenazi [a], Inbar Brosh [a], Shy Shoham [b], Shulamit Levenberg [a], Daniel Razansky [c,d], Amir Rosenthal [e], and Katrien Vandoorne [a]**

[a] *Faculty of Biomedical Engineering, Technion – Israel Institute of Technology, Haifa, 3200003, Israel*
[b] *NYU Langone Health, Tech4Health and Neuroscience Institutes, and Department of Ophthalmology, New York, NY, 10016, United States*
[c] *Faculty of Medicine, University of Zurich, Zurich, 8057, Switzerland*
[d] *Department of Information Technology and Electrical Engineering, ETH Zurich, Zurich, 8093, Switzerland*
[e] *Faculty of Electrical Engineering, Technion – Israel Institute of Technology, Haifa, 3200003, Israel*

**Email:* k.vandoorne@technion.ac.il

I – Materials and Methods;

II – Additional figures;

III– Supplementary references.

I. Methods

Animals. Eight-week-old female C57BL/6J mice (20 ± 2 g, Envigo, Israel) were used for optoacoustic imaging and BALB/c mice were used for optical imaging. Acute systemic inflammation was induced via intraperitoneal injection of lipopolysaccharide (LPS; 0.8 mg/kg, Sigma-Aldrich) 18 hrs prior to imaging. Mice were anesthetized with isoflurane (2.5–2.8% induction, 1–2% maintenance) in oxygen or air and positioned in a stereotaxic skull holder. For intravital imaging, we used FUCCI+ (B6.Cg-Tg(Fucci)610Bsi) transgenic mice (RIKEN) expressing fluorescent cell cycle indicators. HSPCs in the proliferative G1 phase fluoresce (Kusabira Orange: Ex 548 nm, Em 561 nm). All procedures were approved by the Technion Animal Care and Use Committee.

Multi-spectral Optoacoustic Tomography (MSOT). MSOT imaging was performed using a custom tomographic setup[1]. Optical illumination was provided by an OPO laser (SpitLight DPSS EVO 150 OPO, Innolas Laser GmbH), emitting 420–680 nm at up to 100 pulses/s. Optoacoustic signals were captured by a 512-element spherical matrix array transducer (Imasonic SaS, Voray, France) with 10 MHz central frequency and ~75 µm resolution and an effective FOV of 100 mm^2 along the lateral dimensions. Phantoms were prepared by embedding fluorescent magnetic beads (Cospheric, CA) in 0.8% agarose gel (prepared in PBS, w/v). To create a uniform gel matrix, agarose solution was heated until fully dissolved, poured into a 35 mm Petri dish, and allowed to cool slightly until viscous. Fluorescent beads were carefully positioned either on the surface or embedded at desired depths within the gel matrix before full solidification. To prepare the

superficial cranial bone layer, mouse calvaria were harvested immediately post-mortem and, the outer cortical bone layer of the calvaria was carefully dissected away from the inner bone and marrow. The bone layer was rinsed and stored in PBS to maintain hydration until use. The mouse superficial cranial bone layer was placed directly on top of the phantom surface or submerged just beneath the agarose surface. A 3D back-projection algorithm was used for reconstruction [2].

For in vivo MSOT imaging of calvarial bone marrow, the fur overlying the skull was shaved, and mice were secured in a stereotaxic apparatus consisting of two ear bars and an incisor metal bite bar. Ultrasound gel was applied to ensure proper acoustic coupling between the head and the membrane at the base of the coupling water tank in all experiments. Initial imaging validation (n = 6 mice) involved a skin incision to directly expose the calvaria, allowing direct comparison of optoacoustic signals during oxygen and air inhalation. Mice were anesthetized with isoflurane (1.5%), acclimatized to room air for 8 minutes, and underwent a 1-minute baseline air measurement. This was followed by 100% oxygen delivery at 1 L/min via nose cone for 8 minutes, with a 1-minute oxygen baseline measurement, before returning to room air for recovery. Data were recorded continuously and averaged over 1-minute intervals. We subsequently compared this semi-invasive approach to a fully noninvasive imaging method, where only the fur was shaved (n = 9 control, n = 9 LPS-treated). Mice were secured in the stereotaxic holder with ultrasound gel for coupling. Imaging was performed using a wavelength range of 530–584 nm at 2 nm intervals, pulse repetition frequency (PRF) of 10 Hz, one pulse per wavelength, laser excitation energy of 700–900 nJ per pulse, lateral field of view (FOV) of 10 × 10 mm (x–y plane), frequency cutoff of 0.1–6.0 MHz, speed of sound set to 1480 m/s, MSOT gain of 6 dB, and 50 frames per scan (~2.5 minutes per acquisition). The wavelength range of 530–584 nm was chosen due to the strong and distinct absorption features of oxygenated and deoxygenated hemoglobin within this part of the

visible spectrum. These wavelengths provide optimal contrast for accurate spectral unmixing of HbO_2 and Hb, which is essential for quantifying tissue oxygenation. In our model, the calvarial bone marrow lies close to the surface, allowing robust signal detection at these wavelengths. Immediately after noninvasive imaging of healthy control mice and mice with LPS-induced inflammation, blood and femur samples were collected. Physiological parameters were monitored (MouseStat Jr., Kent Scientific).

Custom Matlab scripts reconstructed and generated optoacoustic images from signals acquired at 530–584 nm in 2 nm intervals. Raw data were processed using a model-based reconstruction algorithm to correct light intensity and detector sensitivity variations[3,4], followed by baseline correction to reduce noise. Volumetric images were analyzed as a 4D dataset, with laser pulse energy fluctuations normalized across frames for consistency. A wavelength vector was aligned with image frames to account for acquisition timing and ensure accurate multispectral reconstruction. Spectral unmixing was performed to quantify oxygenated hemoglobin (HbO_2) and deoxygenated hemoglobin (Hb) using a linear least-squares regression approach[5]. Since this process normalizes contributions of Hb and HbO_2 to solve for their relative proportions within each voxel, these values do not represent absolute concentrations, but rather their relative distribution in the tissue. Five regions of interest (ROIs) were analyzed per mouse. Hemoglobin oxygen saturation (sO_2) was calculated as:

$$\mathrm{sO_2(\%)} = \frac{\mathrm{HbO_2}}{\mathrm{Hb} + \mathrm{HbO_2}} \times 100$$

Oxygenation maps display sO_2 values within the interfrontal bone marrow. This analysis included correction for laser energy fluctuations and temporal misalignment between frames, ensuring robust and precise quantification of bone marrow oxygenation.

Ex Vivo MicroCT Imaging. Vascular structures were visualized via aortic perfusion with Microfil (FlowTech Inc.)[6]. MicroCT scans were obtained using a high-resolution microCT scanner (Skyscan 1276, Bruker, Kontich, Belgium) with 55 kV source voltage, 72 μA source current, applied 0.25 mm aluminum filter using a 0.2-degree rotation step, and 8 μm isotropic voxel size before and after decalcification in 0.5 M EDTA. All the resulting projection images were reconstructed using the NRecon software (v.1.7.4.5, Bruker-microCT, Kontich, Belgium) with post-alignment, beam hardening corrections (41%), and a ring artefact reduction. Images were analyzed in CTAn and 3D visualized in CTVox.

CT/MSOT Co-Registration. Calvarial blood vessels were imaged by both MSOT and microCT. Proper alignment of the skull was ensured using anatomical fiducials of the calvaria. Subsequently, MSOT image data were manually aligned with the microCT images to establish the correspondence of hemoglobin signals from MSOT images with the calvarian bone marrow cavities.

Complete Blood Count. Blood was collected via cardiac puncture into EDTA-coated microtubes and analyzed using an automated hematology analyzer (IDEXX ProCyte Dx). Neutrophil percentages were calculated relative to total leukocytes.

Immunohistochemistry. Mice received intravenous albumin-Cy5 (2.5 mg/mouse, SyMO-Chem, Eindhoven) 10 min before sacrifice. For hypoxia analysis, pimonidazole (60 mg/kg, Hypoxyprobe Inc.) was injected 2 hrs prior to euthanasia. Femurs were excised, fixed in 4% paraformaldehyde (PFA), and decalcified for 4 days using MoL Decalcifier (EDTA based decalcifying solution; Milestone, Bergamo, Italy). Skulls (for initial validation) and femurs (for control versus LPS) were transferred into 4% PFA solution decalcified for 4 days using MoL Decalcifier (EDTA based

decalcifying solution; Milestone, Bergamo, Italy), and embedded in paraffin. Decalcification softens the mineralized matrix of bone by removing calcium and other minerals, making it pliable for precise histological sectioning and microscopy analysis without significantly altering its overall thickness. Once soft and pliable, skulls and femurs were paraffinized. Longitudinal sections of the femur with 4 μm thickness were cut and mounted on slides with DAPI. For the skulls, one slide was stained with H&E staining and scanned under light microscope (Slide scanner 250 Flash III, 3D Histech Ltd, Budapest, Hungary). Another adjacent slide was stained with 4',6-diamidino-2-phenylindole (DAPI; Sigma-Aldrich). For the femurs, antigen retrieval was done and subsequent overnight using antibodies targeting endomucin (anti-goat, Novus Biologicals, AF4666), Ki67 (anti-rat, Abcam, ab15580), and separately with FITC-conjugated anti-pimonidazole (HP2-200Kit, Hypoxyprobe Inc.). Secondary antibodies from Thermofischer Scientific (Donkey anti-Rat IgG AF488, A21208; A10040; Donkey anti-Goat IgG AF 680, A21084) were used on femoral sections to enhance the signal, and nuclei were counterstained with DAPI.

Optical Imaging of Bone Marrow Hypoxia Using IVISense Hypoxia CA IX 680 Fluorescent Probe. To ensure optimal optical signal detection, fur was completely using an electric shaver followed by depilatory cream. BALB/c mice were anesthetized and placed on a heated imaging stage during all imaging procedures. Mice received 100 μL (2 nmol) of IVISense Hypoxia CA IX 680 (Revvity, NEV11070) via retroorbital injection. Mice were imaged 24 hours post-injection, the time point determined as optimal for detecting probe accumulation in hypoxic regions. Imaging was performed using preclinical IVIS (IVIS® SpectrumCT, PerkinElmer). The following imaging parameters were applied: excitation at 649 nm and emission at 666 nm.
For 2D fluorescence imaging, mice were positioned such that the region of interest faced the detector. Fluorescence images were acquired and analyzed using the system's native software,

ensuring consistent regions of interest (ROIs) across animals. For ex vivo quantification of fluorescence intensity, the femur, muscle, skull, brain, liver, kidney, spleen, and heart were dissected, and imaged to detect specific fluorescence of IVISense Hypoxia CA IX 680. Fluorescence IVIS imaging data were analyzed with Live Image® 4.5.4 software.

Confocal Microscopy. Ex vivo and intravital imaging was conducted using a CSU-W1 spinning disk confocal microscope (Intelligent Imaging Innovations). For intravital microscopy, mice received OsteoSense 750EX (PerkinElmer) intravenously 24 hrs prior to imaging. $FUCCI^+$ mice were i.v. injected with FITC-albumin (2.5mg/mouse) to visualize bloodvessels. Images were analyzed using ImageJ.

Statistical Analysis. Statistical tests were conducted in Prism 10 (GraphPad). Data normality was assessed via the Shapiro-Wilk test. Wilcoxon matched-pairs signed-rank tests were used for oxygenation comparisons. Mann-Whitney tests analyzed control vs. LPS-treated groups. Pearson correlations assessed oxygenation relationships. Significance was set at $P < 0.05$. Detailed statistical information is in figure legends.

II. Supporting Figures S1-S6

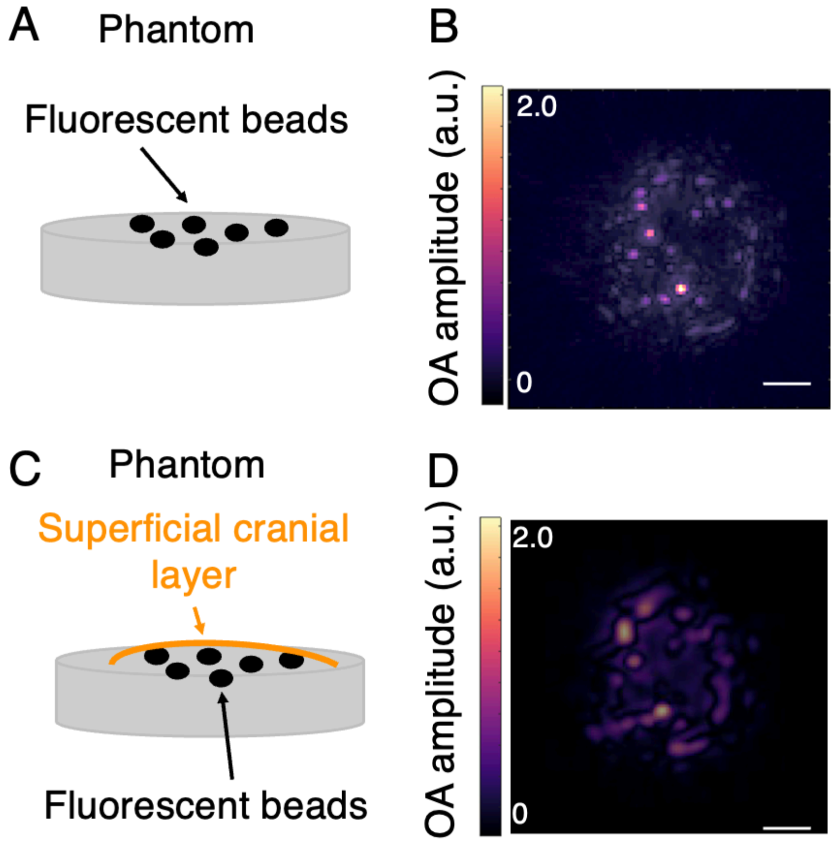

Fig. S1. Optoacoustic imaging of phantom embedded fluorescent beads. (**A**) Schematic of the phantom setup with fluorescent magnetic beads positioned on the surface of 0.8% agarose gel in PBS. (**B**) OA reconstructed image of the phantom showing optical contrast of the beads. (**C**) OA reconstructed image of the phantom with a superficial cranial bone layer placed above the beads. (**D**) OA reconstructed image of beads embedded within the agarose gel, beneath the superficial cranial bone layer. Excitation wavelength: 530 nm. Real-time data acquisition parameters: speed of sound = 1480 m/s, FOV = 10 × 10 mm, frequency filter = 0.1–6.0 MHz. Scale bar = 1 mm.

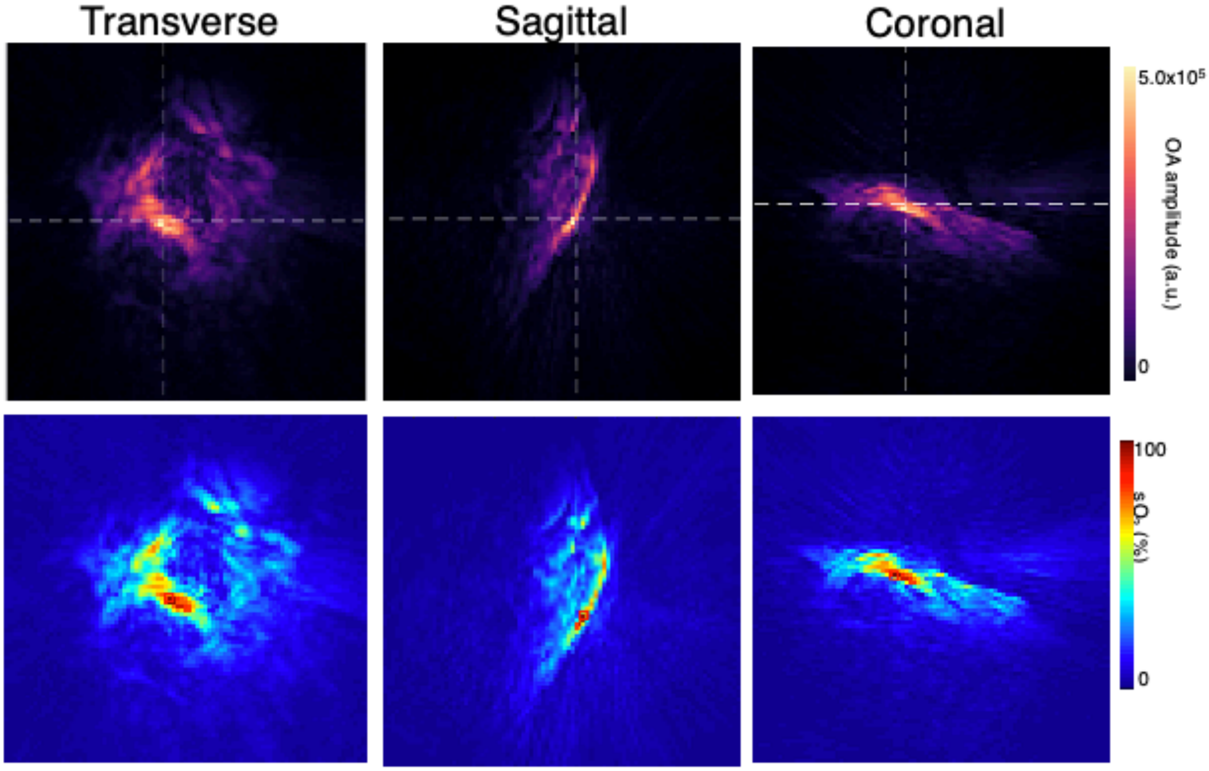

Fig. S2. Three-dimensional (3D) MSOT imaging data showcasing corresponding oxygenated hemoglobin (HbO_2) maps within the calvarial hematopoietic bone marrow of a representative mouse. Dotted lines highlight a region of interest corresponding to the hematopoietic bone marrow.

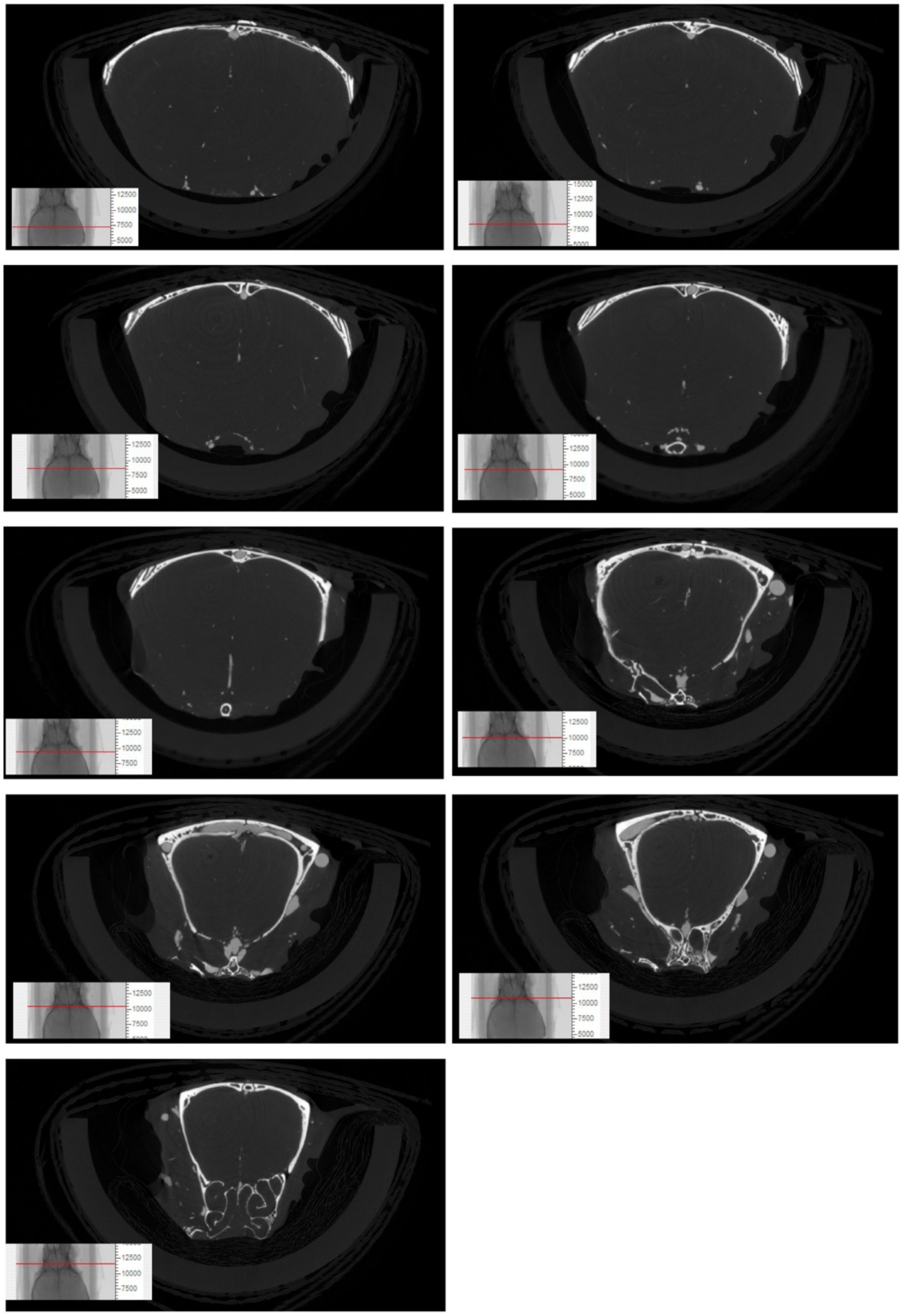

Fig. S3. Separate slices of microCT scans of microfil-injected calvarian bone marrow vessels, and cranium in mice.

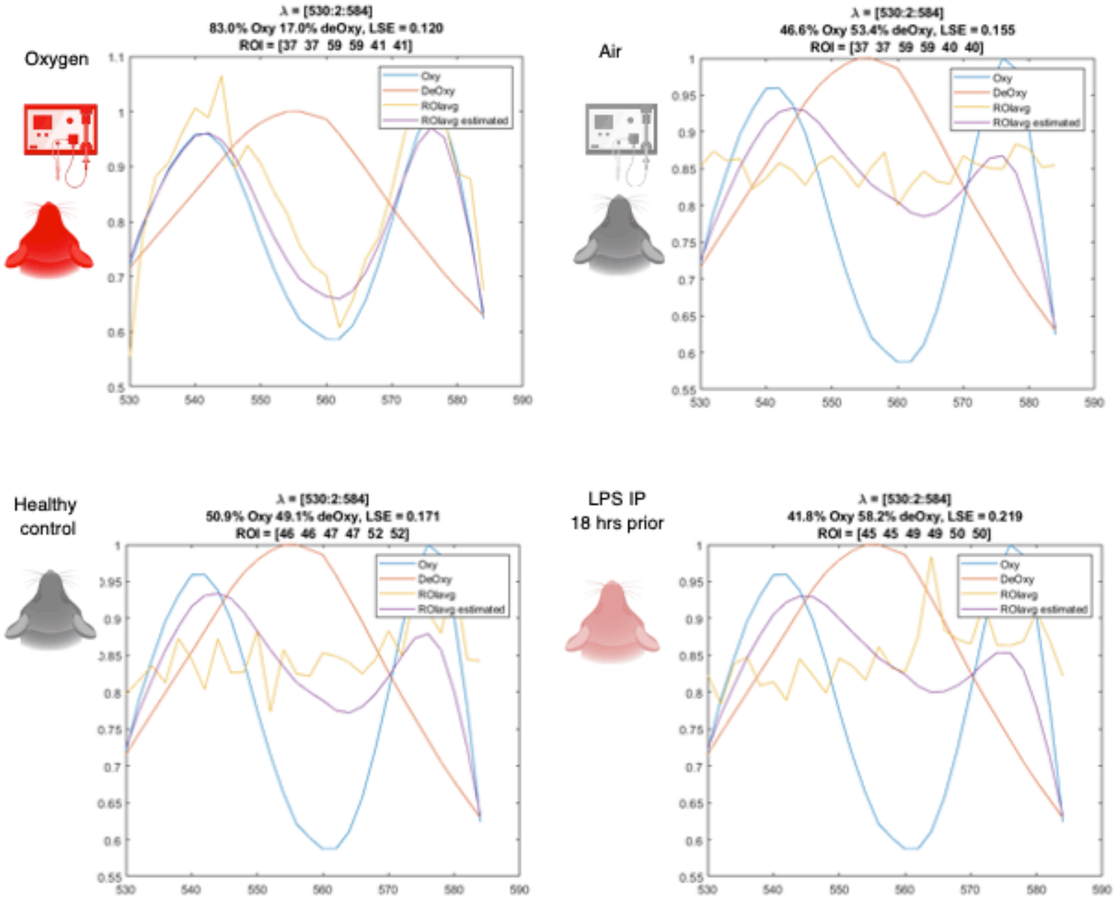

Fig. S4. Absorption spectra of oxygenated (HbO_2) and deoxygenated hemoglobin (Hb), along with the average and estimated signal spectra extracted from the MSOT data. HbO_2 (=Oxy) values for the calvarial hematopoietic marrow in (upper row) mice inhaling either oxygen or air and (lower row) control mice and mice after LPS injection (LPS) at the calvarial hematopoietic marrow.

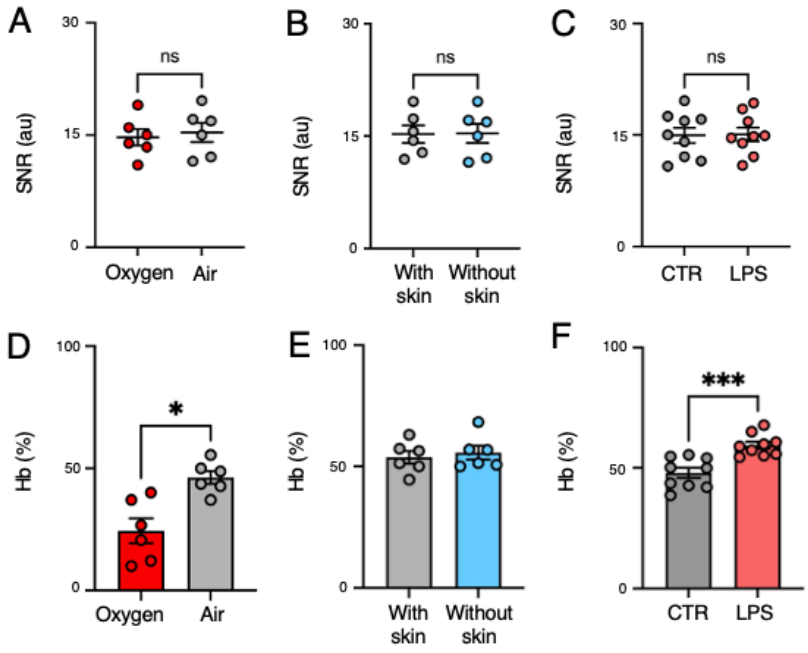

Fig. S5. Signal-to-noise ratio (SNR) of the optoacoustic signal recorded at 560 nm and corresponding deoxygenated hemoglobin (Hb) values under different imaging conditions. **(A)** SNR comparison during oxygen vs. air inhalation. **(B)** SNR comparison in mice imaged noninvasively with the skin intact vs. invasively after skin-flap surgery (with vs. without skin). **(C)** SNR in control (CTR) and LPS-treated mice. **(D)** Hb values during oxygen vs. air inhalation. **(E)** Hb values in mice imaged with and without the skin. **(F)** Hb values in CTR and LPS-treated mice.

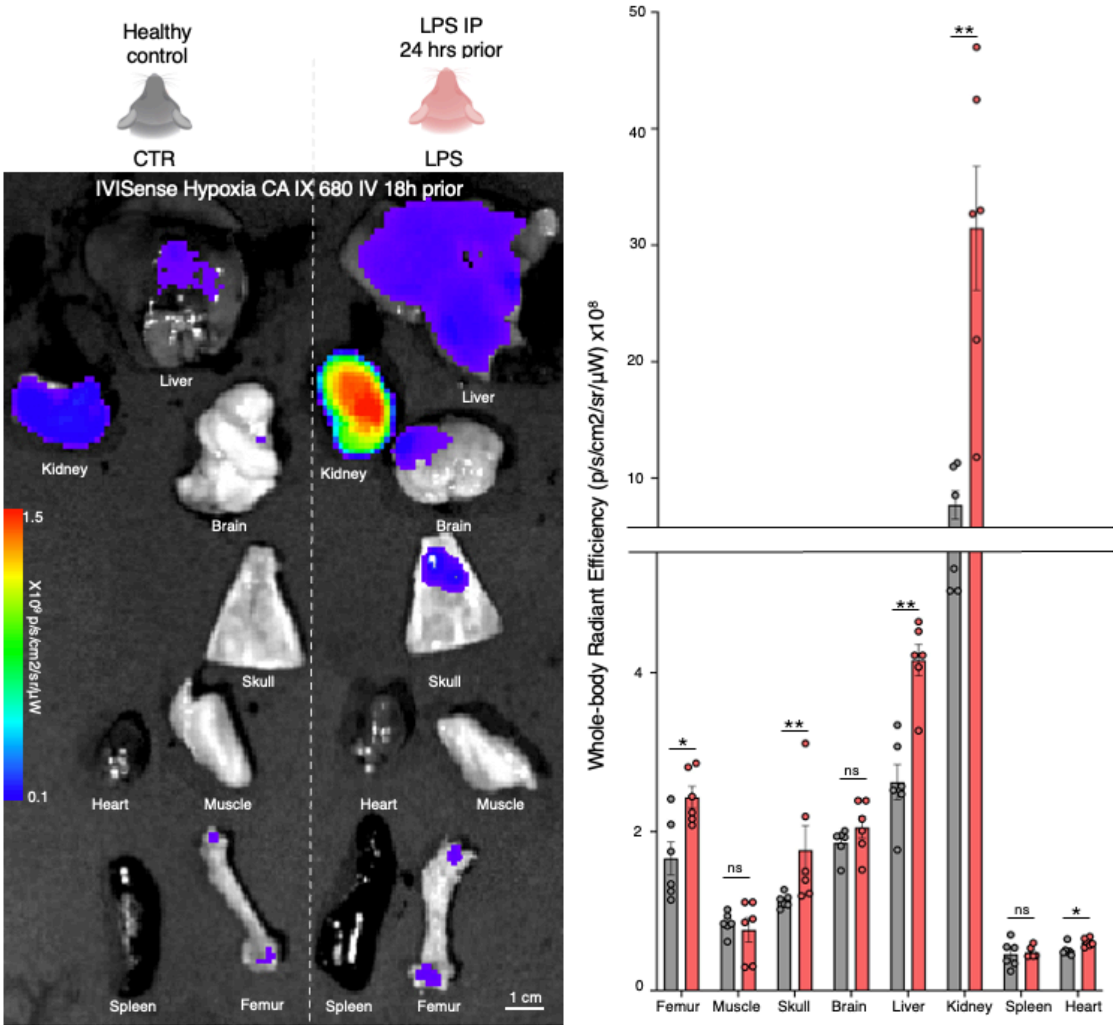

Fig. S6. Ex vivo imaging and quantification of IVISense Hypoxia CA IX 680 uptake. **(A)** Representative ex vivo fluorescence images of isolated organs from control and LPS-treated mice, including femur, muscle, skull, brain, liver, kidney, spleen, and heart. Imaging was performed 18 hours after probe injection and 24 hours after LPS administration (for the LPS-treated group). **(B)** Quantification of radiant efficiency in the indicated organs. LPS-treated mice exhibited significantly elevated probe uptake in both femoral and calvarial bone marrow compared to controls, indicating inflammation-induced hypoxia in hematopoietic compartments. Data are

presented as mean ± SEM. Statistical analysis between two groups per organ was performed using the Mann-Whitney test (*$P < 0.05$, **$P < 0.001$)

III. Supporting References

(1) Deán-Ben, X. L.; Sela, G.; Lauri, A.; Kneipp, M.; Ntziachristos, V.; Westmeyer, G. G.; Shoham, S.; Razansky, D. Functional Optoacoustic Neuro-Tomography for Scalable Whole-Brain Monitoring of Calcium Indicators. *Light Sci Appl* **2016**, *5* (12), e16201–e16201. https://doi.org/10.1038/lsa.2016.201.

(2) Deán-Ben, X. L.; Razansky, D. Optoacoustic Image Formation Approaches—a Clinical Perspective. *Phys. Med. Biol.* **2019**, *64* (18), 18TR01. https://doi.org/10.1088/1361-6560/ab3522.

(3) Deán-Ben, X. L.; Gottschalk, S.; Mc Larney, B.; Shoham, S.; Razansky, D. Advanced Optoacoustic Methods for Multiscale Imaging of in Vivo Dynamics. *Chem. Soc. Rev.* **2017**, *46* (8), 2158–2198. https://doi.org/10.1039/C6CS00765A.

(4) Ding, L.; Luís Deán-Ben, X.; Lutzweiler, C.; Razansky, D.; Ntziachristos, V. Efficient Non-Negative Constrained Model-Based Inversion in Optoacoustic Tomography. *Physics in Medicine & Biology* **2015**, *60* (17), 6733. https://doi.org/10.1088/0031-9155/60/17/6733.

(5) Wood, C.; Harutyunyan, K.; Sampaio, D. R. T.; Konopleva, M.; Bouchard, R. Photoacoustic-Based Oxygen Saturation Assessment of Murine Femoral Bone Marrow in a Preclinical Model of Leukemia. *Photoacoustics* **2019**, *14*, 31–36. https://doi.org/10.1016/j.pacs.2019.01.003.

(6) Redenski, I.; Guo, S.; Machour, M.; Szklanny, A.; Landau, S.; Egozi, D.; Gabet, Y.; Levenberg, S. Microcomputed Tomography-Based Analysis of Neovascularization within Bioengineered Vascularized Tissues. *ACS Biomater. Sci. Eng.* **2022**, *8* (1), 232–241. https://doi.org/10.1021/acsbiomaterials.1c01401.
